# Mechanism of Fluid Reabsorption in Kidney Proximal Tubule: Interplay Between Lateral Na^+^/K^+^‐ATPase and AQP1 and SGLT1 Mediated Water Fluxes

**DOI:** 10.1111/apha.70242

**Published:** 2026-05-19

**Authors:** Erik Hviid Larsen, Jens Nørkær Sørensen

**Affiliations:** ^1^ Department of Biology University of Copenhagen Copenhagen Denmark; ^2^ Department of Wind Energy Technical University of Denmark Lyngby Denmark

## Abstract

**Aims:**

The mechanism of isosmotic water reabsorption in the kidney proximal tubule, with a focus on the interaction between the lateral Na^+^/K^+^‐ATPase, apical water pathways mediated by AQP1 and SGLT1, and paracellular water flow through Claudin‐2.

**Methods:**

A mathematical model of proximal tubular transport was used to compute coupled ion, solute, and water fluxes. The model included apical ENaC and a full electrogenic Na^+^/K^+^‐ATPase formulation that incorporated its electromotive force, *E* ^pump^, as a thermodynamic constraint linked to ATP hydrolysis at the pump site.

**Results:**

Published cellular cation concentrations indicate metabolic stress in excised tubules, providing biophysical rationale for interpreting enhanced NHE3 activity and proton secretion in isolated proximal tubules as consequences of reduced *ΔG* ^ATP^, while modeling apical Na^+^ entry under non‐stressed conditions by ENaC. Active Na^+^ transport generated a slightly hyperosmotic and hyperbaric lateral intercellular space, driving fluid efflux across the interspace basement membrane. Without ion recirculation, the absorbed fluid remained hyperosmotic. Isosmotic reabsorption therefore required ion recirculation between serosal fluid and the lateral intercellular space. SGLT1‐mediated glucose uptake redistributed water flow between AQP1, SGLT1 and the paracellular pathway, whereas total water reabsorption remained closely linked to active Na^+^ transport, consistent with experiments.

**Conclusion:**

Proximal tubular water reabsorption is not explained by passive osmotic equilibration alone, but emerges from thermodynamic coupling between active Na^+^ transport, water permeability pathways, and regulated ion recirculation. The proximal tubule therefore functions as an ATP‐consuming epithelial fluid pump that maintains isosmotic reabsorption by using additional metabolic energy to convert initially hyperosmotic absorbate into isosmotic reabsorbed fluid.

## 1 Introduction

In the late 19th century, the Scottish physiologist E. Waymouth Reid published what was at the time an astonishing finding: epithelial fluid uptake takes place in the absence of a driving force for water [1]. Since his study on the isolated frog skin and small intestine, it was shown that this mode of water transport is coupled to a simultaneous uptake of NaCl [2, 3, 4], thereby ‘explaining the water absorption as a secondary effect due to its dependence on active salt transport’ [3]. The renal proximal tubule is a classical epithelium that reabsorbs fluid essentially isosmotically [5]. In transporting epithelia, the Na^+^/K^+^‐ATPase is expressed predominantly in the lateral membranes [6, 7, 8]. Computational modeling of the proximal nephron predicts that the Na^+^/K^+^‐ATPase produces a slightly hyperosmotic and hyperbaric lateral intercellular space. If the reflection coefficient of tight junction (*σ* ^tj^) is larger than that of the interspace basement membrane (*σ* ^ibm^), at transepithelial osmotic equilibrium fluid flows from luminal to peritubular space [9]. Introducing the Na^+^/K^+^‐pump fluxes in biophysical analyses of epithelial function requires mathematical expression of its coupling to cellular energy metabolism. To this end we follow Ussing's concept of the pump electromotive force, *E* ^*pump*^ [10, 11], which represents the free energy change of ATP hydrolysis (*ΔG* ^*ATP*^) in the immediate vicinity of the pump sites. The water pathways of proximal tubule are mediated by aquaporin, AQP1 [12, 13] and the luminal Na^+^‐glucose cotransporters, SGLT1 [14, 15, 16, 17]. An additional water uptake occurs via junctional Claudin‐2 [18], which is permeable also to Na^+^ and K^+^ [19]. The present study focuses on epithelial water fluxes mediated by these pathways and their dependence on the activity of the Na^+^/K^+^‐pump. Identification of the ion and water pathways described above has relied on experimental studies on biological preparations. However, the coupled interactions among Na^+^/K^+^‐pump flux, transmembrane ion fluxes, and water movement along their respective pathways are best analyzed by mathematical modeling, which permits quantitative evaluation of the system as an integrated whole [9, 20].

## 2 Mathematical Model of the Tubular Epithelium

**FIGURE 1:**
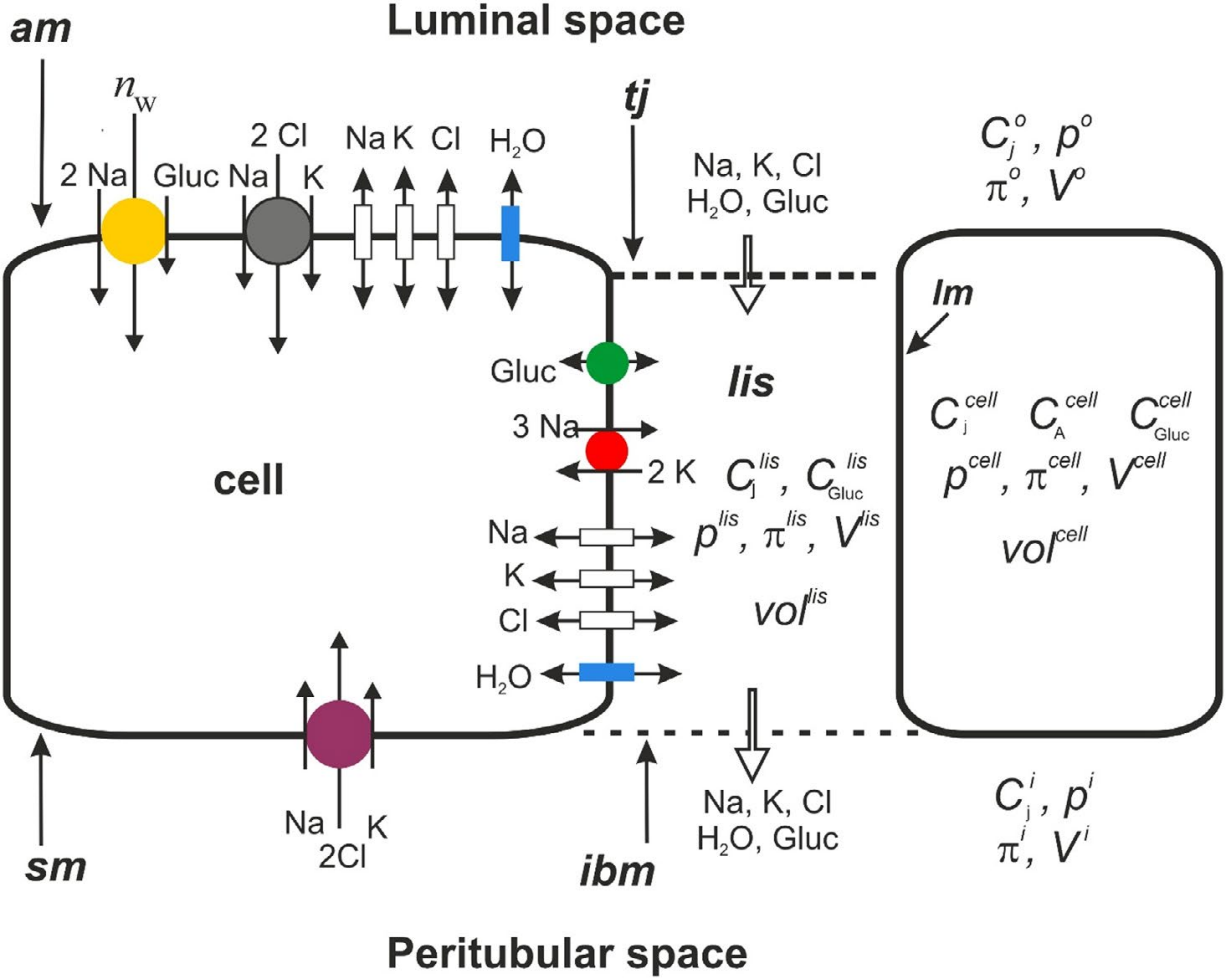
Functional organization of the minimalistic kidney tubule with three membrane domains, apical (*am*), lateral (*lm*), and serosal (*sm*), respectively. Tight junction (*tj*) and interspace basement membrane (*ibm*) provide the entrance to and the exit from, respectively, the lateral intercellular space (*lis*). Note that water and solutes transported across *am* from the luminal space into the cell are transported across *lm* into *lis* for exiting the epithelium via *ibm* together with water and solutes entering *lis* through *tj*. Luminal space is also denoted ‘outer solution’. Similarly, serosal solution is synonymous with ‘inner solution’. The transport pathways indicated are explained in text Section 2.

The principal outline of our mathematical model has been presented previously [9, 20, 21] and is restated here with updated notes and references. The model describes the proximal tubule as a minimalistic epithelium symmetrically bathed by well‐stirred saline with concentrations of Na^+^, K^+^, Cl^−^, and glucose, which do not change during the individual simulation. The epithelium contains well‐stirred cellular and paracellular compartments, which have access to both the outside and the inside bath confined by five membranes; apical (*am*), serosal (*sm*), lateral (*lm*), tight junction (*tj*), and interspace basement membrane (*ibm*), see Figure 1. All model variables are expressed per unit area of the apical membrane.

Micropuncture studies demonstrated that bicarbonate is preferentially reabsorbed in the early proximal tubule (S1–S2), so that the tubular fluid entering the proximal straight tubule (S3) contains very little bicarbonate and is correspondingly enriched in chloride [22, 23]. Although the model formally represents the proximal straight tubule by restricting mobile ions to Na^+^, K^+^, and Cl^−^, the resulting linkage between pump electromotive force, intracellular Na^+^ and K^+^ concentrations, and the requirement for isosmotic fluid reabsorption are segment‐independent determinants of proximal tubular transport.

### 2.1 Cellular Solute Flux Equations

Electrodiffusion fluxes are computed by the GHK constant field‐equation with associated integral conductance equation,

(1a) $$J_{j}=\left(\frac{z_{j}\mathrm{FV}}{\mathrm{RT}}P_{j}\right)\frac{C_{j}^{\left(I\right)}\exp\left[z_{j}\mathrm{FV}/\left(\mathrm{RT}\right)\right]-C_{j}^{\left(\mathrm{II}\right)}}{\exp\left[z_{j}\mathrm{FV}/\left(\mathrm{RT}\right)\right]-1}$$

(1b) $$G_{j}=\left(\frac{{\left(z_{j}F\right)}^{2}V}{\mathrm{RT}}P_{j}\right)\frac{C_{j}^{I}\exp\left[z_{j}\mathrm{FV}\right]-C_{j}^{\mathrm{II}}}{\left(\exp\left[z_{j}\mathrm{FV}/\mathrm{RT}\right]-1\right)\left(V-E_{j}\right)}$$

*P*_*j*_ is the ion permeability and *V* is the potential difference across the membrane. I = *lumen* and II = *cell* for the apical membrane, I = *cell* and II = *lis* for the lateral membrane, and I = *cell* and II = *serosal* (peritubular) space for the serosal membrane. Willmann et al. [24] showed that both the S2 and the S3 segment contain PCR products for the α, β and γ subunit, respectively, of the sodium ion channel, ENaC. They demonstrated small conductance (12 pS) amiloride‐sensitive channels of high P_Na_/P_K_ selectivity to be present in the luminal membrane, which are the typical characteristics of ENaC. Rainer Greger and his associates pointed out that the contribution of Na^+^ channel‐mediated absorption to total proximal Na^+^ absorption is “probably small”. This study from 1997 confirms the previous patch clamp study by Gögelein and Greger characterizing Na^+^ selective channels in the apical membrane of rabbit pars recta [25]. That is, in the two thoroughly studied mammalian species—rat and rabbit—the kidney proximal tubule expresses functional ENaC in the apical membrane.

The ion transporters in the apical membrane (Figure 1) do not capture that in vitro the apical Na^+^ uptake occurs predominantly via electroneutral Na/H exchange mediated by the electroneutral NHE3 [26, 27]. As discussed in detail below, bioenergetic stress in isolated nephrons leads to excess cytosolic proton generation requiring cellular elimination. The resulting acidification down‐regulates electrogenic amiloride‐inhibitable Na^+^ uptake via ENaC, thereby forcing electroneutral Na^+^ uptake via NHE3, which explains the observed combination of high NHE3 activity and reduced ENaC activity in excised proximal tubules [28, 29, 30].

A component of K^+^ reabsorption in proximal tubule is passive and driven by paracellular solvent drag [19, 31], which is included in our model. A small transcellular active component, which is not energized by a K‐H ATPase [32], is mechanistically unresolved. The significance of uphill K^+^ uptake is to obtain a K^+^ concentration of reabsorbed fluid close to that of the extracellular fluid. As a simplifying model assumption (not substantiated experimentally), apical K^+^ uptake was parameterized as Na^+^ coupled cotransport:

(2) $$J_{j}^{\mathrm{am},\mathrm{NaK}2\mathrm{Cl}}=r\cdot K^{\mathrm{am},\mathrm{NaK}2\mathrm{Cl}}\left[C_{\mathrm{Na}}^{\left(I\right)}\cdot C_{K}^{\left(I\right)}\cdot {\left(C_{\mathrm{Cl}}^{\left(I\right)}\right)}^{2}-C_{\mathrm{Na}}^{\left(\mathrm{II}\right)}\cdot C_{K}^{\left(\mathrm{II}\right)}\cdot {\left(C_{\mathrm{Cl}}^{\left(\mathrm{II}\right)}\right)}^{2}\right]$$

where *r* = 1 for Na^+^ and K^+^ and *r* = 2 for Cl^−^. The application of Equation (2) enables electroneutral secondary active Cl^−^ uptake, which is consistent with cellular Cl^−^ accumulation via an electroneutral Na^+^‐coupled apical mechanism in Necturus [33, 34, 35].

The proximal tubule absorbs most of the glucose in the glomerular filtrate [36]. In the kidney tubule's S2‐ and S3 segments, glucose is coupled to Na^+^ uptake via SGLT1 with a stoichiometry of 2 Na^+^:1 glucose [15]. SGLT1 also functions as a low‐conductance water pathway that couples Na^+^ and sugar of fixed stoichiometry and varying coupling‐ratio between glucose‐ and H_2_O molecules [37, 38]. Driving forces for glucose uptake across the luminal membrane are the transmembrane glucose concentration gradient, the transmembrane Na^+^ concentration gradient, and the apical membrane potential, *V* ^*am*^. To simplify the integration, we assumed that one glucose and two Na^+^ molecules pass through a ‘constant‐field membrane’ in unity as a divalent particle in accordance with a recent model of SGLT1 transport [39]. With $n_{W}^{\text{SGLT}1}$ indicating the ratio of water molecules associated the glucose uptake, the following three instantaneously coupled flux equations apply,
(3a)

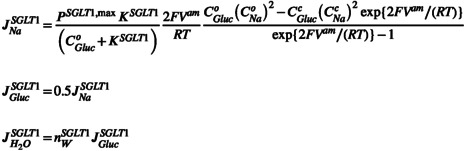




Zeuthen and coworkers discussed the evidence of a common membrane pathway for sodium ions, glucose‐ and water molecules [38]. It is emphasized that the system of coupled equations does not presuppose any specific molecular mechanism underlying SGLT1‐mediated water uptake across the luminal membrane of proximal tubule. Even if future studies may demonstrate that coupling between solute fluxes and water flow is not instantaneous, this would not alter the conclusions of the present steady‐state analysis. Saturation kinetics of glucose uptake from tubular fluid is introduced by a luminal‐glucose‐dependent SGLT1 permeability,

(3b) $$P^{\text{SGLT}1}=P^{\text{SGLT}1,\max}\left[K^{\text{SGLT}1}/\left(C_{\text{Gluc}}^{o}+K^{\text{SGLT}1}\right)\right]$$

**FIGURE 2:**
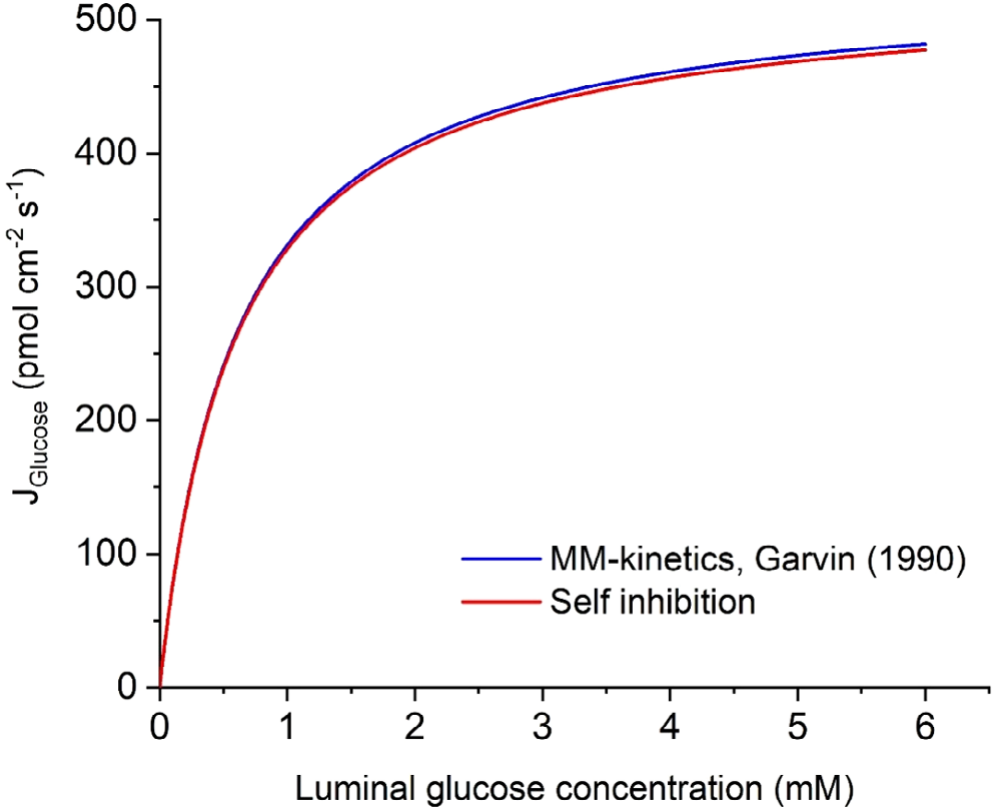
Saturation kinetics of glucose absorption in the proximal straight tubule (pars recta; S3 segment). Experimental data from rat kidney were fitted with the Michaelis‐Menten equation, *J*_*Gluc*_ = 20 × $C_{\text{Gluc}}^{o}$/(0.6 + $C_{\text{Gluc}}^{o}$) pmol·mm^−1^·min^−1^ [40]. For comparison, the relationship was recalculated to express the glucose flux per luminal surface area assuming a cylindrical lumen with a diameter of 20 μm, *J*_*Gluc*_ = 530 × $C_{\text{Gluc}}^{o}$/(0.6 + $C_{\text{Gluc}}^{o}$), blue curve labeled, “MM‐kinetics, Garvin (1990)”. The red curve labeled “self‐inhibition” was calculated from Eqs. 3a‐3b for luminal glucose concentrations ranging from 0 to 6 mM using the following values, $C_{\mathrm{Na}}^{o}=146$ mM, $C_{\mathrm{Na}}^{c}=10$ mM, $C_{\text{Gluc}}^{c}=8$ mM, *V* ^apical^ = −60 mM, *K* ^*SGLT1*^ = 1.0 mM, *T* = 310 K; $P^{\text{SGLT}1,\max}$ was set to yield a maximal glucose flux of 530 pmol·cm^−2^·s^−1^.

**FIGURE 3:**
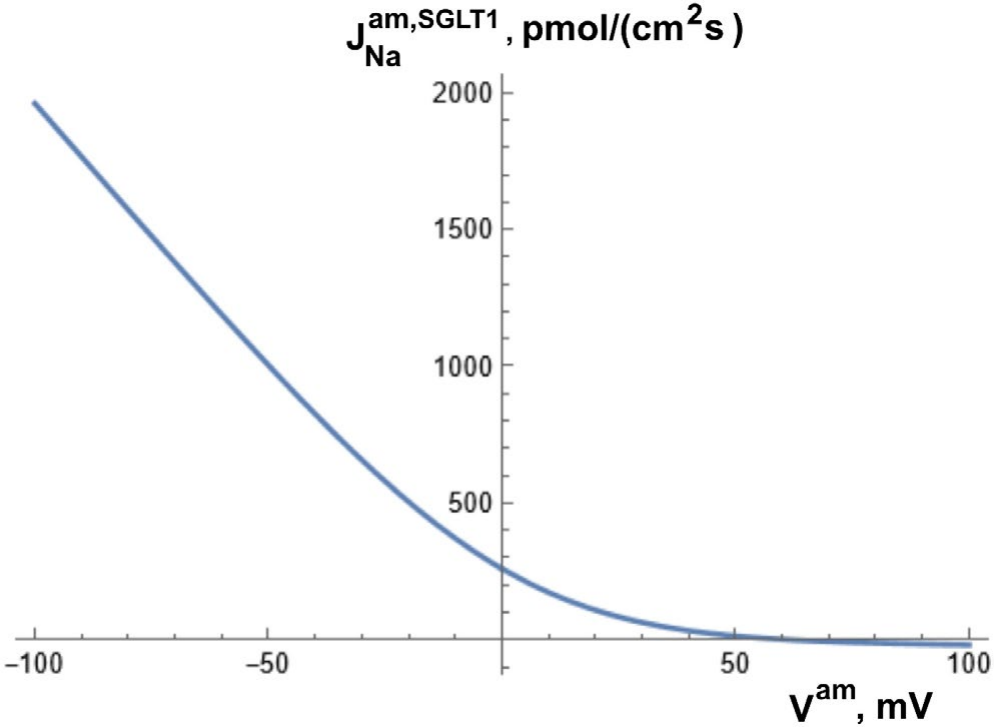
Voltage dependence of the Na^+^ flux in SGLT1 mathematically given by Equation (3a) of Section 2. The near‐linear relationship between −40 and −100 mV of *V* ^*am*^ accords with experiment [41].

Figure 2 compares the concentration dependence of glucose uptake in proximal straight tubule with predictions from the Michaelis–Menten formalism [40] and from the self‐inhibition mechanism (Equation 3b), respectively, which in Equation (3a) explicitly links glucose‐coupled Na^+^ flux to membrane potential and intracellular Na^+^ and glucose concentrations. The near complete overlap of the two curves indicates that the self‐inhibition mechanism reproduces classical Michaelis–Menten saturation behavior. Voltage‐clamp studies of cloned SGLT1 [41] revealed near‐linear voltage‐dependence of SGLT1 currents in the voltage range between −40 and −100 mV, which is reproduced by the above Equation (3a), see Figure 3. Independent of the shape of the current–voltage relationship, the reversal potential, $V_{\mathrm{rev}}^{\mathrm{am},\text{SGLT}1}$ is that of the electrically charged Na^+^ flux carried by SGLT1,

(3c) $$V_{\mathrm{rev}}^{\mathrm{am},\text{SGLT}1}=\frac{\mathrm{RT}}{2F}\ln\frac{C_{\text{Gluc}}^{o}{\left(C_{\mathrm{Na}}^{o}\right)}^{2}}{C_{\text{Gluc}}^{c}{\left(C_{\mathrm{Na}}^{c}\right)}^{2}}$$

In the physiological range of membrane potentials, the integral conductance of SGLT1 is,

(3d) $$\begin{array}{c} G_{\mathrm{Na}}^{\text{SGLT}1}=\frac{2{\mathrm{FV}}^{\mathrm{am}}}{\mathrm{RT}}\frac{P^{\text{SGLT}1,\max}K^{\text{SGLT}1}}{\left(C_{\text{Gluc}}^{o}+K^{\text{SGLT}1}\right)}\frac{C_{\text{Gluc}}^{o}{\left(C_{\mathrm{Na}}^{o}\right)}^{2}-C_{\text{Gluc}}^{c}{\left(C_{\mathrm{Na}}^{c}\right)}^{2}\exp\left\{2{\mathrm{FV}}^{\mathrm{am}}/\left(\mathrm{RT}\right)\right\}}{\left(\exp\left\{2{\mathrm{FV}}^{\mathrm{am}}/\left(\mathrm{RT}\right)\right\}-1\right)\left(V^{\mathrm{am}}-V_{\mathrm{rev}}^{\mathrm{am},\text{SGLT}1}\right)} \end{array}$$

Exit flux of glucose via GLUT1 [42] from cell to *lis* is obtained by Stein's equation for a symmetrical, saturating carrier [43],

(4) $$J_{\text{Gluc}}^{\mathrm{lm}}=J_{\text{Gluc}}^{\mathrm{lm},\max}\frac{K_{\text{Gluc}}^{\mathrm{lm}}\left(C_{\text{Gluc}}^{c}-C_{\text{Gluc}}^{\mathrm{lis}}\right)}{\left(K_{\text{Gluc}}^{\mathrm{lm}}+C_{\text{Gluc}}^{c}\right)\left(K_{\text{Gluc}}^{\mathrm{lm}}+C_{\text{Gluc}}^{\mathrm{lis}}\right)}$$

The Na^+^/K^+^‐ATPase [44] is confined to the basolateral membrane [6], particularly the lateral membrane lining the intercellular clefts, where it drives solutes accumulation within the lateral intercellular space. The rate of active cation fluxes saturates at large concentration of Na^+^ in the cell, $C_{\mathrm{Na}}^{c}$ and the concentration of K^+^ in the lateral intercellular space, $C_{K}^{\mathrm{lis}}$. The rate of pumping of the two cations across the lateral membrane (*lm*) with a stoichiometry of 3Na^+^/2 K^+^ is also a function of the electric work performed in moving one charge across the membrane per pump cycle, which depends on membrane potential, $V^{\mathrm{lm}}=\psi^{\text{cell}}-\psi^{\mathrm{lis}}$ [45]. With *E* ^*pump*^ denoting the electromotive force of the Na^+^/K^+^ pump, the active cation fluxes are given by the following set of equations [9, 20, 21]

(5a) $$\begin{array}{c} \begin{array}{l} J_{\mathrm{Na}}^{\mathrm{lm},\text{pump}}=P^{\mathrm{lm},\text{pump}}{\left(\frac{C_{\mathrm{Na}}^{c}}{K_{\mathrm{Na}}^{\mathrm{lm},\text{pump}}+C_{\mathrm{Na}}^{c}}\right)}^{3}{\left(\frac{C_{K}^{\mathrm{lis}}}{K_{K}^{\mathrm{lm},\text{pump}}+C_{K}^{\mathrm{lis}}}\right)}^{2}\\ \left(V^{\mathrm{lm}}+E^{\text{pump}}\right)\\ J_{K}^{\mathrm{lm},\text{pump}}=-\frac{2}{3}J_{\mathrm{Na}}^{\mathrm{lm},\text{pump}} \end{array} \end{array}$$

Here, $P^{\mathrm{lm},\text{pump}}$ has dimension of mol·s^−1^ ·V^−1^ per unit area of apical membrane. For kidney, $K_{\mathrm{Na}}^{\mathrm{lm},\text{pump}}$ = 3.4 mM and $K_{K}^{\mathrm{lm},\text{pump}}$ = 0.75 mM, respectively [46]. With the stoichiometry of the pump being 3 Na^+^: 1 ATP [46] and introducing the Faraday, the pump current is,

(5b) $$\begin{array}{c} I^{\mathrm{lm},\text{pump}}=F\frac{P^{\mathrm{lm},\text{pump}}}{3}{\left(\frac{C_{\mathrm{Na}}^{c}}{K_{\mathrm{Na}}^{\mathrm{lm},\text{pump}}+C_{\mathrm{Na}}^{c}}\right)}^{3}{\left(\frac{C_{K}^{\mathrm{lis}}}{K_{K}^{\mathrm{lm},\text{pump}}+C_{K}^{\mathrm{lis}}}\right)}^{2}\\ \left(V^{\mathrm{lm}}+E^{\text{pump}}\right) \end{array}$$

When expressed in terms of the pump conductance $G^{\mathrm{lm},\text{pump}}$, the equations take the form,

$$I^{\mathrm{lm},\text{pump}}=G^{\mathrm{lm},\text{pump}}\left(V^{\mathrm{lm}}+E^{\text{pump}}\right)$$

(5c) $$G^{\mathrm{lm},\text{pump}}=F\frac{P^{\mathrm{lm},\text{pump}}}{3}{\left(\frac{C_{\mathrm{Na}}^{c}}{K_{\mathrm{Na}}^{\mathrm{lm},\text{pump}}+C_{\mathrm{Na}}^{c}}\right)}^{3}{\left(\frac{C_{K}^{\mathrm{lis}}}{K_{K}^{\mathrm{lm},\text{pump}}+C_{K}^{\mathrm{lis}}}\right)}^{2}$$

We introduce the reversal potential of the Na^+^/K^+^‐ATPase, $V_{\mathrm{rev}}^{\mathrm{lm},\text{pump}}$, which implies that the pump can operate in both forward and reverse directions. Such reversibility was indicated by showing incorporation of radioactive phosphate into ATP at the expense of downhill cation movement in red cells [47]. Equations (5) imply that $V_{\mathrm{rev}}^{\mathrm{lm},\text{pump}}$ is equal in magnitude but opposite in sign to *E* ^*pump*^, i.e., $V_{\mathrm{rev}}^{\mathrm{lm},\text{pump}}=-E^{\text{pump}}$. In the physiological range of membrane potentials, Equation (5b) generates pump currents that are linearly dependent on *V* ^*lm*^, which accords with experiment [45].

### 2.2 Paracellular Flux Equations

Paracellular solvent drag on diffusible ions is computed by Hertz's equation [48, 49] applied to a porous membrane with a nonzero reflection coefficient, *σ*_*j*_, of ion *j* [50, 51],

(6) $$\begin{array}{c} J_{j}=\left(\frac{z_{j}\mathrm{FV}}{\mathrm{RT}}P_{j}+J_{V}\left(1-\sigma_{j}\right)\right)\\ \frac{C_{j}^{\left(I\right)}\exp\left[z_{j}\mathrm{FV}/\left(\mathrm{RT}\right)\right]\exp\left[J_{V}\left(1-\sigma_{j}\right)/P_{j}\right]-C_{j}^{\left(\mathrm{II}\right)}}{\exp\left[z_{j}\mathrm{FV}/\left(\mathrm{RT}\right)\right]\exp\left[J_{V}\left(1-\sigma_{j}\right)/P_{j}\right]-1} \end{array}$$

For the tight junction membrane, *I* = *o*, while II = *lis*. For the interspace basement membrane, I = *lis* and II = the peritubular space. Equation (6) assumes a symmetric pore with reflection coefficient ($\sigma_{j}$) and partition coefficient (β) related by $\sigma_{j}=1–\beta$ [52]. *J*_*V*_ is the volume flow from compartment I (lumen or *lis*) to II (*lis* or peritubular space). Equation (6) applies to Na^+^ and K^+^ passing the paracellular tight junctions through the water permeable Claudin‐2 [18, 19, 53], and is applied for solute pathways of *ibm*. Claudin‐17/10a are permeable to small anions, but not to water [18, 54]; thus, the junctional Cl^−^ flux is calculated by the equation for simple electrodiffusion (cf. Equation (1)). Solvent drag on sucrose and other electroneutral molecules in the kidney proximal tubule [55, 56] makes it plausible that the electroneutral glucose is also submitted to paracellular transport by frictional interactions with water, which agrees with the recent study of small intestine [57]. This flux may correspond with the component of glucose uptake in the proximal tubule designated ‘moderate glucose leak’ [58]. Thus, the paracellular glucose flux across the tight junction (*tj*) and interspace basement membrane (*ibm*) are computed by [50]

(7a) $$J_{\text{Gluc}}^{\mathrm{tj}}=J_{V}^{\mathrm{tj}}\left(1-\sigma_{\text{Gluc}}^{\mathrm{tj}}\right)\frac{C_{\text{Gluc}}^{o}\exp\left[J_{V}^{\mathrm{tj}}\left(1-\sigma_{\text{Gluc}}^{\mathrm{tj}}\right)/P_{\text{Gluc}}^{\mathrm{tj}}\right]-C_{\text{Gluc}}^{\mathrm{lis}}}{\exp\left[J_{V}^{\mathrm{tj}}\left(1-\sigma_{\text{Gluc}}^{\mathrm{tj}}\right)/P_{\text{Gluc}}^{\mathrm{tj}}\right]-1}$$

(7b) $$J_{\text{Gluc}}^{\mathrm{ibm}}=J_{V}^{\mathrm{ibm}}\left(1-\sigma_{\text{Gluc}}^{\mathrm{ibm}}\right)\frac{C_{\text{Gluc}}^{\mathrm{lis}}\exp\left[J_{V}^{\mathrm{ibm}}\left(1-\sigma_{\text{Gluc}}^{\mathrm{ibm}}\right)/P_{\text{Gluc}}^{\mathrm{lis}}\right]-C_{\text{Gluc}}^{s}}{\exp\left[J_{V}^{\mathrm{ibm}}\left(1-\sigma_{\text{Gluc}}^{\mathrm{ibm}}\right)/P_{\text{Gluc}}^{\mathrm{lis}}\right]-1}$$

### 2.3 Water Flows in Aquaporins

In agreement with the cloned aquaporins of proximal tubules [59], we assume a reflection coefficient of unity for water flow through cell membranes. With *i* denoting the individual element or molecule, the equations for the respective volume flows per unit area of apical membrane are [9, 20]

(8a) $$J_{V}^{\mathrm{am}}=L_{p}^{\mathrm{am}}\left\{\mathrm{RT}\left(\sum C_{i}^{c}-\sum C_{i}^{o}\right)+\left(p^{o}-p^{c}\right)\right\}$$

(8b) $$J_{V}^{\mathrm{lm}}=L_{p}^{\mathrm{lm}}\left\{\mathrm{RT}\left(\sum C_{i}^{\mathrm{lis}}-\sum C_{i}^{c}\right)+\left(p^{c}-p^{\mathrm{lis}}\right)\right\}$$

(8c)

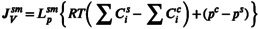

As the osmotic permeability of the individual SGLT1 channel is at most 1% of that of a single aquaporin molecule (AQP1) independent of the presence of glucose [38], we assume that the small *osmotic* water flow through SGLT1 is included in Equation (8a). The equations for water flow through membranes, *tj* and *ibm*, delimiting the lateral intercellular space from luminal and peritubular solutions, respectively, include reflection coefficients [9, 20],

(9a) $$J_{V}^{\mathrm{tj}}=L_{p}^{\mathrm{tj}}\left\{\mathrm{RT}\left(\sum \sigma_{i}^{\mathrm{tj}}\left(C_{i}^{\mathrm{lis}}-C_{i}^{o}\right)\right)+\left(p^{o}-p^{\mathrm{lis}}\right)\right\}$$

(9b) $$J_{V}^{\mathrm{ibm}}=L_{p}^{\mathrm{ibm}}\left\{\mathrm{RT}\left(\sum \sigma_{i}^{\mathrm{ibm}}\left(C_{i}^{s}-C_{i}^{\mathrm{lis}}\right)\right)+\left(p^{\mathrm{lis}}-p^{s}\right)\right\}$$

The reflection coefficients of tight junctions are obtained from the literature, $\sigma_{\mathrm{Na}}^{\mathrm{tj}}=\sigma_{K}^{\mathrm{tj}}=0.7$ and $\sigma_{\mathrm{Cl}}^{\mathrm{tj}}=0.45$ [60], while for the large electroneutral glucose, we assume $\sigma_{\text{Gluc}}^{\mathrm{tj}}=0.8$. The computations to be presented are not sensitive to this choice as long as $\sigma_{\text{Gluc}}^{\mathrm{tj}}>>\sigma_{\text{Gluc}}^{\mathrm{ibm}}$. In general, fluid absorption requires that the reflection coefficient of the interspace basement membrane is lower than that of the tight junction. Here, we assume, $\sigma_{i}^{\mathrm{ibm}}$ = 0.03, which results in hydrostatic pressures of the lateral intercellular space to be about 1.001 atm. In the literature, the hydraulic conductance, *L*_*p*_, is translated to the osmotic permeability, *P*_*f*_. With the molar volume of water indicated by ${\bar{V}}_{W}$, *L*_*p*_ and *P*_*f*_ are related by [52],

(10) $$P_{f}=\frac{{\mathrm{RTL}}_{p}}{{\bar{V}}_{W}}$$

### 2.4 Electroneutrality

With the mean valence of nondiffusible anions in the cell denoted, *z*_*A*_ , electroneutrality requires Equation (11) and (12) to be fulfilled

(11) $$C_{A}^{c}=\left(C_{\mathrm{Na}}^{c}+C_{K}^{c}-C_{\mathrm{Cl}}^{c}\right)/z_{A}$$

(12) $$C_{\mathrm{Cl}}^{\mathrm{lis}}=C_{\mathrm{Na}}^{\mathrm{lis}}+C_{K}^{\mathrm{lis}}$$

If *I* ^*clamp*^ is the transepithelial clamping current and *I*_*j*_ is the current carried by *j* (*j* = Na^+^, K^+^, or Cl^−^) through the membrane indicated by superscript, the mathematical solution would have to obey the following requirement:

(13) $$I^{\text{clamp}}=I_{\mathrm{Na}}^{\mathrm{am}}+I_{\mathrm{Na}}^{\mathrm{tj}}+I_{K}^{\mathrm{am}}+I_{K}^{\mathrm{tj}}+I_{\mathrm{Cl}}^{\mathrm{am}}+I_{\mathrm{Cl}}^{\mathrm{tj}}$$

Here, *I* ^*clamp*^ = 0 defines the mathematical solution containing the transepithelial potential difference, *V* ^*trans*^. All simulations are for the tubular epithelium in the open circuit mode.

### 2.5 Hydrostatic Pressure and Intraepithelial Volumes

To obtain the hydrostatic pressures of the cell and *lis*, we apply the following compliance model [9],

(14) $$p^{c}=\left(\mu^{\mathrm{am}}p^{o}+\mu^{\mathrm{lm}}p^{\mathrm{lis}}+\mu^{\mathrm{sm}}p^{s}\right)/\left(\mu^{\mathrm{am}}+\mu^{\mathrm{lm}}+\mu^{\mathrm{sm}}\right)$$

in which *μ* is the compliance factor of the membrane indicated. With *lis* volume in the absence of fluid flow indicated by *Volis* ^*lis,ref*^, the volume of *lis*, *Vol* ^*lis*^ is given by,

(15) $${\mathrm{Vol}}^{\mathrm{lis}}={\mathrm{Vol}}^{\mathrm{lis},\mathrm{ref}}\left[1+\mu^{\mathrm{lm}}\left(p^{\mathrm{lis}}-p^{c}\right)\right]$$

Number of cells per unit area of apical membrane and the amount of nondiffusible anions per cell are *D* ^*c*^ and *M*_*A*_, respectively. With $C_{A}^{c}$ being the dependent variable, the volume of the functional syncytium is,

(16) $${\mathrm{Vol}}^{c}=D^{c}M_{A}/C_{A}^{c}$$

### 2.6 Electrical‐Circuit Analysis and Sign Conventions

Because the transepithelial potential difference is a function of current flow in cellular and paracellular pathways, for a given set of independent variables, *V* ^*trans*^ is computed after the solution to the above set of equations is obtained. Our method makes use of the resistance of each of the five membranes of the equivalent bridge circuit, *R* ^*m*^ (*m* = 1–5) obtained from the summation of chord conductances of individual ion pathways, i.e., Equation (1b) for ion channels, Equation (3c) for SGLT1, and Equation (5b) for the Na^+^/K^+^‐pump. To simulate a step change in the transepithelial current, *ΔI* ^trans^, Kirchhoff's rules are applied to set up five simultaneous linear equations,

(17a) $$I_{1}R_{1}+I_{3}R_{3}-I_{4}R_{4}=0$$

(17b) $$I_{3}R_{3}+I_{5}R_{5}-I_{2}R_{2}=0$$

(17c) $$I_{2}+I_{5}-\Delta I^{\text{trans}}=0$$

(17d) $$I_{1}-I_{2}-I_{3}=0$$

(17e) $$I_{4}-I_{3}-I_{5}=0$$

Currents, *I*_*n*_ (*n* = 1–5), flowing through the five resistors were obtained by the *Solve routine* of *Mathematica*,

(18a) $$\begin{array}{c} I_{1}=-\frac{-\left(R_{2}R_{4}+R_{3}R_{4}+R_{3}R_{5}+R_{4}R_{5}\right)}{R_{1}R_{2}+R_{1}R_{3}+R_{2}R_{3}+R_{2}R_{4}+R_{3}R_{4}+R_{1}R_{5}+R_{3}R_{5}+R_{4}R_{5}}\\ \Delta I^{\text{trans}} \end{array}$$

(18b) $$\begin{array}{c} I_{2}=\Delta I^{\text{trans}}\\ +\frac{\left(R_{1}R_{3}+R_{2}\left(R_{1}+R_{3}+R_{4}\right)\right)}{-R_{1}R_{3}-R_{1}R_{4}-R_{2}\left(R_{1}+R_{3}+R_{4}\right)-R_{5}\left(R_{1}+R_{3}+R_{4}\right)}\Delta I^{\text{trans}} \end{array}$$

(18c) $$\begin{array}{c} I_{3}=-\frac{\left(R_{2}R_{4}+R_{1}R_{5}\right)}{R_{1}R_{2}+R_{1}R_{3}+R_{2}R_{3}+R_{2}R_{4}+R_{3}R_{4}+R_{1}R_{5}+R_{3}R_{5}+R_{4}R_{5}}\\ \Delta I^{\text{trans}} \end{array}$$

(18d) $$I_{4}=-\frac{-\left(R_{1}R_{2}-R_{1}R_{3}-R_{2}R_{3}-R_{1}R_{5}\right)}{R_{1}R_{2}+R_{1}R_{3}+R_{2}R_{3}+R_{2}R_{4}+R_{3}R_{4}+R_{1}R_{5}+R_{3}R_{5}}\Delta I^{\text{trans}}$$

(18e) $$\begin{array}{c} I_{5}=-\frac{\left(R_{1}R_{3}+R_{2}\left(R_{1}+R_{3}+R_{4}\right)\right)}{-R_{1}R_{3}-R_{3}R_{4}-R_{2}\left(R_{1}+R_{3}+R_{4}\right)-R_{5}\left(R_{1}+R_{3}+R_{4}\right)}\\ \Delta I^{\text{trans}} \end{array}$$

The transepithelial potential difference is now calculated by:

(19) $$V^{\text{trans}}=I_{1}R_{1}+I_{2}R_{2}\;\left(=I_{4}R_{4}+I_{5}R_{5}\right)$$

Fluxes directed from lumen to cell and to *lis*, from cell to peritubular bath and to *lis*, and from *lis* to peritubular bath have a positive sign. Electrical potentials are referenced with respect to the peritubular space (serosal solution) i.e., *ψ* ^*s*^ ≡ 0.

### 2.7 Numerical Methods

The transport equations for water and solutes are as follows

(20) $$\frac{d\bar{V}}{\mathrm{dt}}=\sum_{m}J_{\bar{V}}$$

(21) $$\frac{d\left(\bar{V}\cdot C_{S}\right)}{\mathrm{dt}}=\sum_{m}J_{S}$$

$\bar{V}$ denotes the volume of the cell or the lateral intercellular space, while $J_{\bar{V}}$ and $J_{S}$ denote the water and solute fluxes through the various membranes, where *m* indicates the membrane (*m* = 1–5). The left‐hand sides are zero at the steady state, while the study of time‐dependent behavior requires Equation (23) and (24) to be simulated. To solve the equations in time, we apply second‐order accurate, three‐point backward difference schemes (Taylor expansion) as follows,

(22) $$\frac{1}{2\Delta t}\left[3{\bar{V}}^{\left(n\right)}-4{\bar{V}}^{\left(n-1\right)}+{\bar{V}}^{\left(n-2\right)}\right]=\sum_{j}J_{\bar{V}}^{\left(n\right)}$$

(23) $$\frac{1}{2\Delta t}\left[3{\left(\bar{V}\cdot C_{S}\right)}^{\left(n\right)}-4{\left(\bar{V}\cdot C_{S}\right)}^{\left(n-1\right)}+{\left(\bar{V}\cdot C_{S}\right)}^{\left(n-2\right)}\right]=\sum_{m}J_{S}^{\left(n\right)}$$

The index *n* refers to time *t* ^*n*^, and Δ*t* is the time step, such that *t* ^*n*^ = *t* ^*n*−1^ + Δ*t*. Thus, the equations are solved for all variables with index *n* at time *t* = *t* ^*n*^, leaving the remaining terms known from former time steps. The equations are solved together with the above equations for electroneutrality and the compliance model. The strongly coupled nonlinear equations were solved for machine accuracy by a conventional iterative Newton–Raphson method. In forming the Jacobian matrix, rather than analytical differentiations, we employed a simple difference scheme.

### 2.8 Independent Variables

Appendices 1 and 2 list the variables of our model of proximal tubule S3 segment given in MKSA units, which in the text are converted to units used in physiological literature. Solute permeabilities and kinetic constants were chosen for simulating physiological values of ion concentrations and fluxes [61], while the hydraulic conductances were obtained from literature [62, 63, 64].

## 3 Results

### 3.1 Quantitative Analysis of Intraepithelial Ion‐ and Water Turnover of Proximal Tubule

Our quantitative treatment of ion and water transport across the tubular epithelium is based on the classical pump‐leak concept. The turnover at the lateral Na^+^/K^+^‐pump and the permeability of the dissipative membrane pathways govern the cellular and paracellular fluxes and the concentration of solutes in cell and lateral intercellular space. To study the dependence of solute concentrations and volumes of intraepithelial compartments on the turnover at the Na^+^/K^+^‐pump we introduce the pump's electromotive force (Ussing) [10, 11], here denoted *E* ^*pump*^ and mathematically presented in Equation (5a) in Section 2 above. In cells exposed to well aerated solutions the free energy of ATP hydrolysis, *∆G* ^ATP^ is about −58 kJ/mol [65, 66, 67, 68, 69]. Recalling that the reversal potential, $V_{\mathrm{rev}}^{\mathrm{lm},\text{pump}}$ of the Na^+^/K^+^‐pump current is equal to in magnitude but opposite in sign to *E* ^*pump*^, with pump stoichiometry of 3Na^+^:2 K^+^:1ATP per pump cycle and mitochondria positioned near Na^+^/K^+^‐pumps [70], in well‐aerated tubules $V_{\mathrm{rev}}^{\mathrm{lm},\text{pump}}$ ≈ −58.10^6^/3*F* = −200 mV. This is in close agreement with the upper‐limit estimate of the pump electromotive force of 200 mV in frog skin reported by Eskesen and Ussing using pre‐steady‐state flux‐ratio analysis [11], and with the corresponding estimate of 205 mV obtained in toad skin from Larsen's electric circuit analysis of amiloride reduced active Na^+^ currents [71]. The kidney's specific metabolic rate is surpassed only by the heart's [72, 73] reflecting the very high rate of metabolic energy‐demanding reabsorption of electrolytes, metabolites and water from the glomerular filtrate, which in human totals about 180 L per day. Vertebrate cells typically contain about 140 mM K^+^ and about 10 mM Na^+^ [74]. In contrast, published concentrations in isolated proximal tubules show unusual ranges: $C_{K}^{c}$, 113 mM in rat [75], 68 mM in rabbit [76], and 70 mM in Necturus [77]; $C_{\mathrm{Na}}^{c}$, 17.5 mM in rat [78], and 44 mM in rabbit [79]. These measurements indicate that excised kidney tubules generally exhibit reduced cytosolic ATP/ADP ratio and therefore reduced free energy available for biochemical work, *in casu* the electrochemical work of the Na^+^/K^+^‐pump. For quantitative investigation (*conf*. our previous study [20]) we applied our mathematical model to compute effects of reduced *E* ^*pump*^ on steady state intracellular concentrations of Na^+^ and K^+^; the re‐calculations based on more realistic cation concentrations of the reference state are listed in Table 1.

**TABLE 1:** Effect of the free energy of ATP hydrolysis (*ΔG* ^*ATP*^) on the reversal potential of the 3Na^+^/2 K^+^ pump current ($V_{\mathrm{rev}}^{\mathrm{lm},\text{pump}}$), on cellular cation concentrations ($C_{\mathrm{Na}}^{\text{cell}}$ and $C_{K}^{\text{cell}}$), and on the lateral membrane potential (*V* ^*cell*^), as computed with the epithelial model described in Section 2.

*∆G* ^*ATP*^ (kJ/mol)	$V_{\mathrm{rev}}^{\mathrm{lm},\text{pump}}$ (mV)	$C_{\mathrm{Na}}^{\text{cell}}$ (mM)	$C_{K}^{\text{cell}}$ (mM)	*V* ^*cell*^ (mV)
−58.0	−200	9.87	136	−75.6
−52.2	−180	10.6	120	−65.0
−46.4	−160	13.9	116	−64.4
−40.6	−140	20.4	110	−63.0
−35.2	−120	35.1	95.8	−59.4
−29.0	−100	60.2	71.5	−52.2

It is seen that the concentration of the two cations strongly depends on the energy supply via the lateral Na^+^/K^+^‐pump (columns 3 and 4). Thus, it is indicated that kidney tubules excised for laboratory experiments suffer from severe energetic constraints caused by insufficient oxygen supply, which would cause accumulation of metabolites such as lactic acid and hydrogen ions. This is a crucial point, as it provides a logical explanation for the very large apical Na^+^/H^+^ exchange flux mediated by NHE3 [26, 80], accompanied by the proton‐suppressed Na^+^ uptake by ENaC [24, 25]. Thus, the above analysis of published intracellular cation concentrations (Table 1) suggests that the model calibrations preferentially should be based on normal physiological (“textbook”) values rather than values reported from laboratory studies of oxygen‐deficient preparations.

### 3.2 Time and Voltage Dependence of Glucose Stimulated Water Uptake at Transepithelial Equilibrium

**FIGURE 4:**
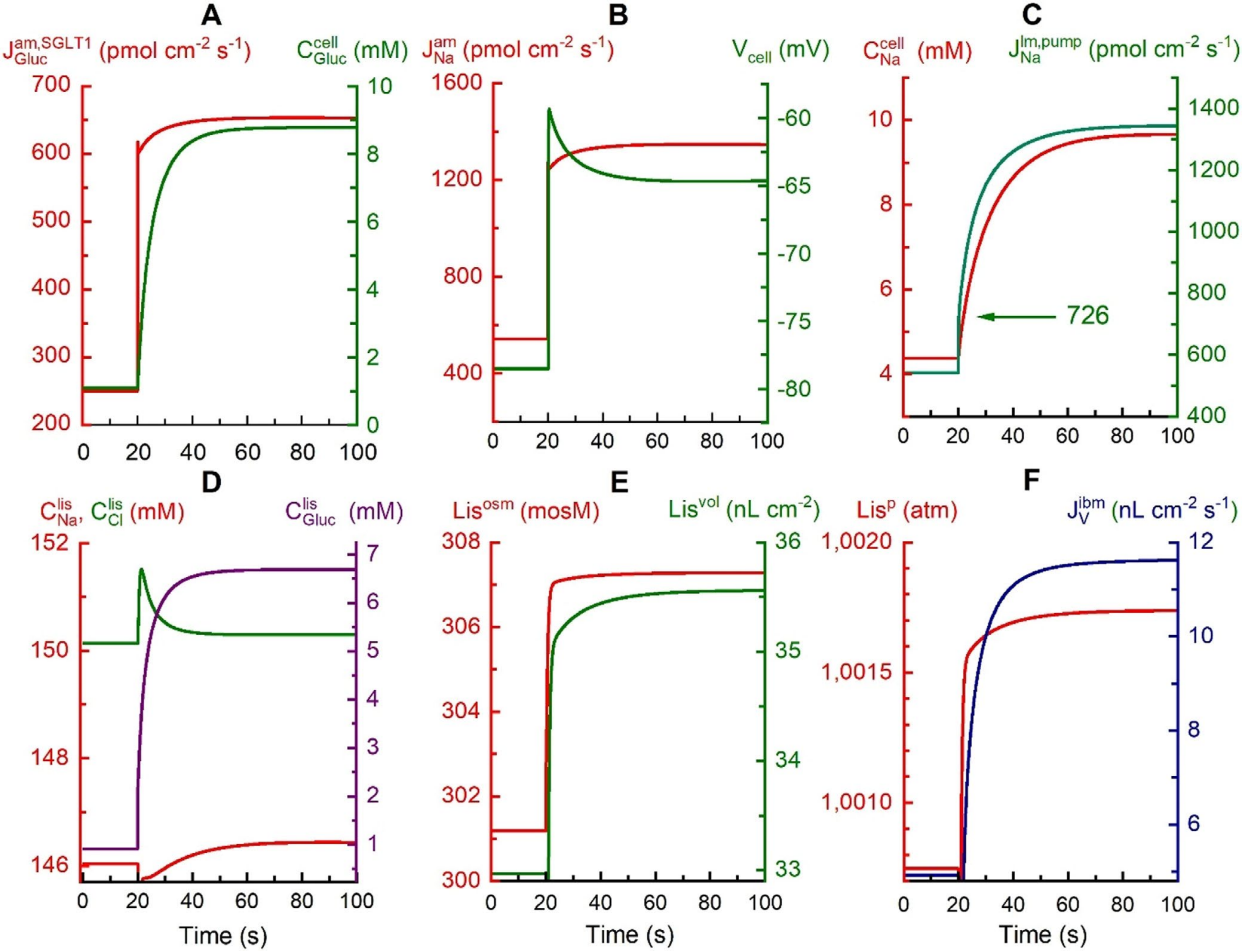
(A–F) Response of physiologically relevant epithelial variables to exponential increase (τ = 0.001 s) in the glucose concentration from 1 to 5 mM on both sides. The number ‘726’ indicated in (C) (green curve) is the instantaneous pump flux resulting from the voltage dependence of the lateral Na^+^/K^+^ pump flux (Equation 5a). For more details, see text.

For elucidating the driving forces for water flows and ion‐ and glucose fluxes across the proximal tubule at transepithelial osmotic equilibrium, we exposed the model epithelium to a monoexponential increase in bilateral glucose concentration from 1.0 to 5.0 mM (τ = 0. 001 s). The resulting time dependent response of relevant physiological variables are given in Figure 4A–F.

Figure 4A: The perturbation protocol initiated a fast increase in the flux of glucose into the cell, $J_{\text{Gluc}}^{\mathrm{am}}$ (Equation 3) from 250 to 651 pmol·cm^−2^·s^−1^, which raised its glucose concentration from 1.88 to 8.7 mM during the first minute after the exposure.

Figure 4B: The tight coupling of glucose flux with the charge carrying sodium flux (Equation 5a) increased the cellular Na^+^ uptake from 541 to 1337 pmol·cm^−2^·s^−1^ resulting in time‐dependent cell depolarization, instantaneously from −78.5 to −59.4 = −19.1 mV (right hand graph).

Figure 4C: The near‐instantaneous membrane depolarization stimulated the voltage dependent Na^+^/K^+^‐pump flux, $J_{\mathrm{Na}}^{\text{pump}}$ (Equation 5), from 541 to 726 pmol·cm^−2^·s^−1^, corresponding to an inward pump current, $\Delta I_{\mathrm{Na}}^{\text{pump}}$ = *F*·(726–541)·10^−6^/3 = 5.95 μA·cm^−2^. The subsequent increase in $J_{\mathrm{Na}}^{\text{pump}}$ is caused by the trivial concentration dependent stimulation of $J_{\mathrm{Na}}^{\text{pump}}$, entangled however, by the concomitant changes in *V*_cell_ and the charge carrying flux through SGLT1 (Equation 3a).

Figure 4D: Prior to increasing the external glucose concentrations, ion concentrations of the lateral intercellular space resemble those of the external baths, $C_{\mathrm{Na}}^{\mathrm{lis}}$ = 146.0 mM, $C_{K}^{\mathrm{lis}}$ = 4.1 mM (not shown), and $C_{\mathrm{Cl}}^{\mathrm{lis}}$ = 150.1 mM, with the glucose concentration being smaller than that of the bathing solutions, $C_{\text{Gluc}}^{\mathrm{lis}}$ = 0.91 mM. Following perturbation, at time = 100 s the solute concentrations given by the model are, $C_{\mathrm{Na}}^{\mathrm{lis}}$ = 146.3 mM, $C_{K}^{\mathrm{lis}}$ = 3.90 mM (not shown), $C_{\mathrm{Cl}}^{\mathrm{lis}}$ = 150.2 mM, and $C_{\text{Gluc}}^{\mathrm{lis}}$ = 6.7 mM, respectively. While the intracellular glucose concentration exceeds that of the bathing solutions because of secondary active uptake, the glucose concentration in the lateral intercellular space is lower than in the cell, since glucose transport from cell to this space is passive (Equation 4).

Figure 4E: While the requirement of electroneutrality (Equation 12) governs the accumulation of electrolytes in *lis*, the lateral membrane's compliant constant (μ^lm^) and the interspace membrane's reflection coefficients (*σ* ^ibm^) determine how much the volume and the hydrostatic pressure of *lis* increase, Equation (14) and (15). With reflection coefficients of 0.03 (Appendix 2), the *lis* volume increases from 33 to 35.5 nL·cm^−2^ with an associated increase in the hydrostatic pressure of the lateral intercellular space, *Lis* ^*p*^, see Figure 4F. The assumption of *σ* ^ibm^ = 3 × 10^−2^ implies that the solutes contribute measurably to the osmotic gradient across the interspace basement membrane. Were *σ* ^ibm^ as low as 1 × 10^−4^, any resulting volume change would be undetectable [20].

Figure 4F: The concomitant increase in hydrostatic pressure of cell and *lis* causes *Lis* ^*p*^ to increase from 1.00 074 to 1.00 173 atm, which increased the fluid flow across the interspace basement membrane from 4.9 to 11.5 nL·cm^−2^·s^−1^ (t = 100 s).

### 3.3 The Problem of Isosmotic Reabsorption

**FIGURE 5:**
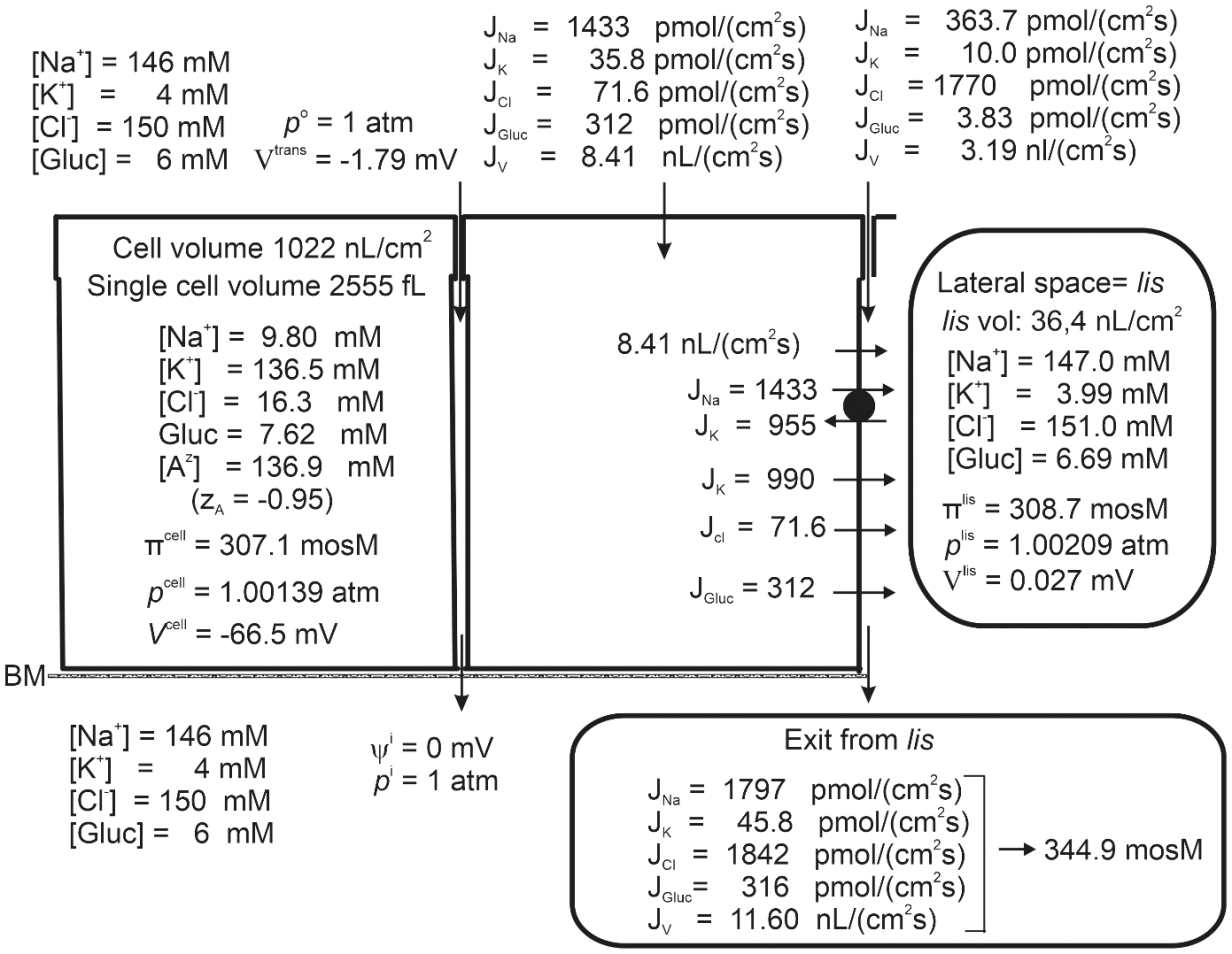
Model epithelium at steady state exposed on both sides to (mM), 146 Na^+^, 4 K^+^, 150 Cl^−^, and 6 glucose. All cellular and paracellular fluxes are given together with intraepithelial values of concentrations, osmolarity, hydrostatic pressure, and electrical potential. Note that the fluid leaving the epithelium (“Exit from *lis*”) is hyperosmotic to bathing solutions (344.9 vs. 306.0 mosM).

**FIGURE 6:**
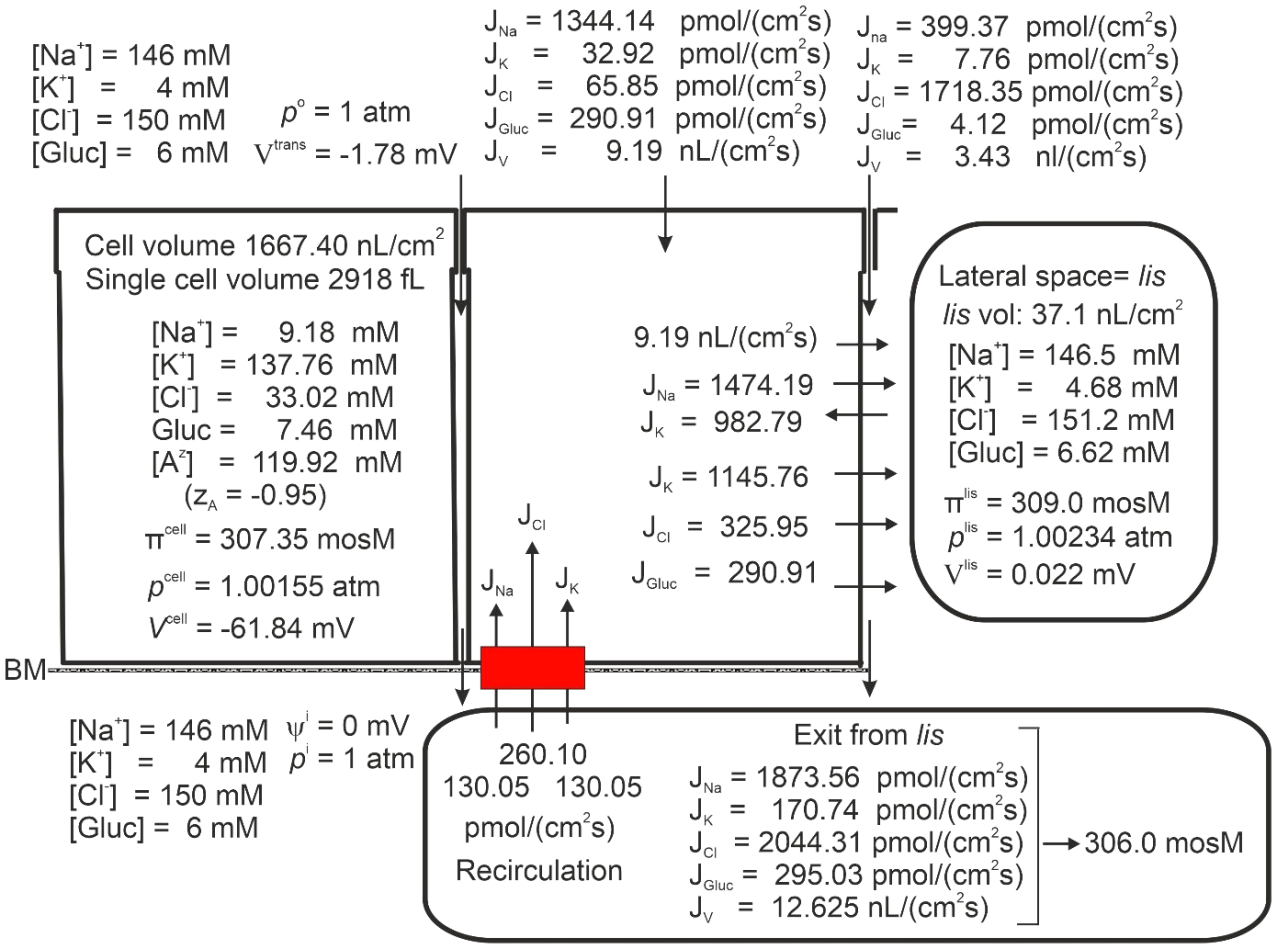
Effect of activating the 1Na^+^:2Cl^−^:1K^+^ cotransporter in serosal membrane for obtaining isosmotic transport by ion‐recirculation. Exit from *lis* across interspace basement membrane now becomes isosmotic to bathing solutions, 306 mosM. However, intracellular Cl^−^ is raised from 16.3 to 33.02 mM with an associated cell volume increase from 1022 to 1667 nL cm^−2^ (compare Figures 5 and 6).

**FIGURE 7:**
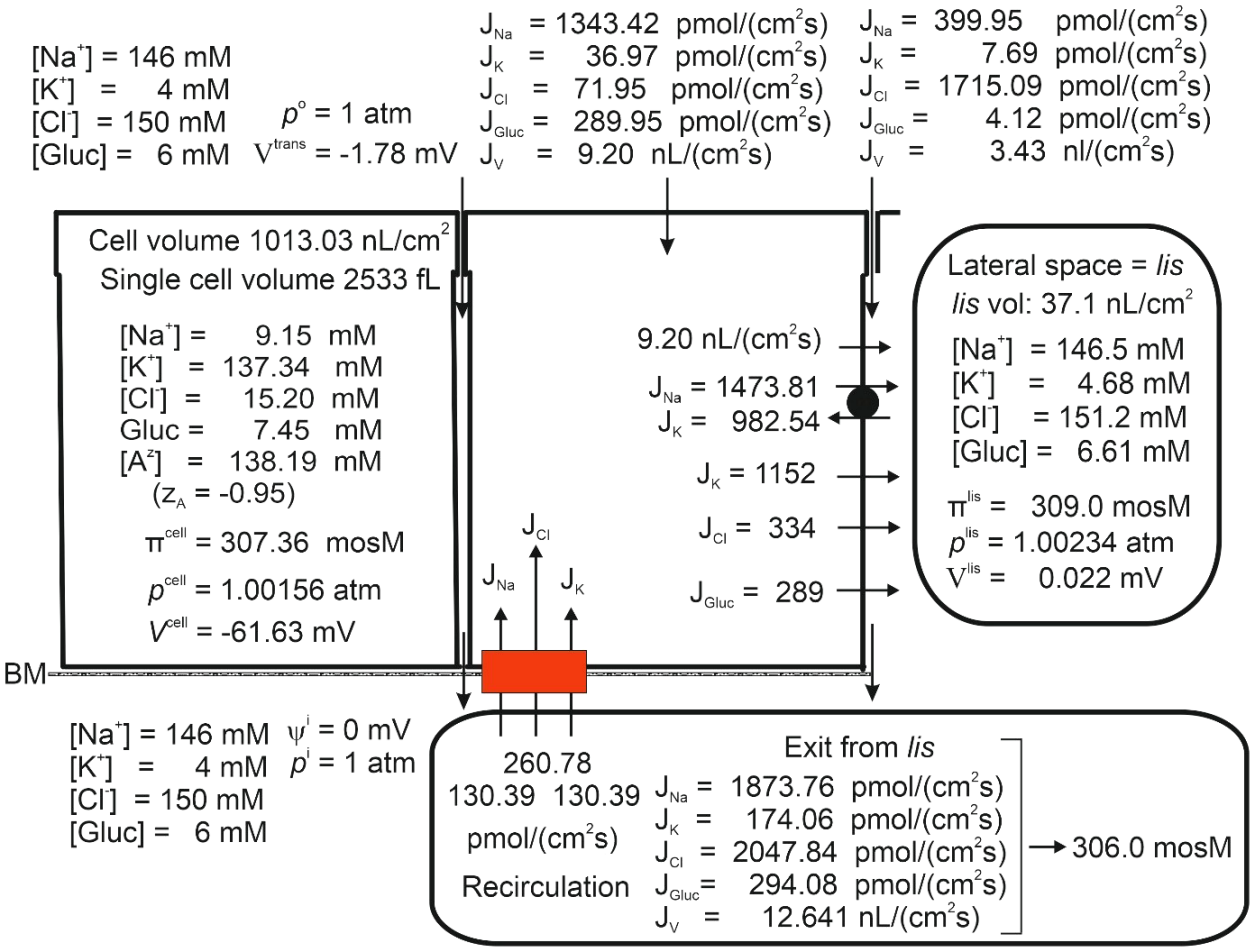
By “VRAC activation,” that is, increasing the Cl^−^ permeability of the lateral membrane, isosmotic transport is maintained, but with return of $C_{\mathrm{Cl}}^{\text{cell}}$ and cell volume into their physiological ranges (compare Figures 5 and 7).

Fluid absorption along the proximal renal tubule leaves the osmolarity of the luminal fluid unchanged, indicating that the absorbed fluid is isosmotic to luminal fluid both at regular osmotic conditions [5, 81, 82] and during water‐ and anti‐diuresis [83]. Figure 5 shows the steady state composition of cell‐ and intercellular fluid compartments with 6 mM peritubular‐ and luminal glucose in physiological saline. The osmolarity of the absorbed fluid is here 345 mosM, that is, 12.7% hyperosmotic to the peritubular fluid of 306 mosM. Notably, the osmolarities of the external solution, cell water, and lateral intercellular space are separated by only small differences (306, 307.1, and 308.7 mosM, respectively). This is a robust result, reproducing our previous analysis of markedly greater fluid reabsorption in the proximal convoluted tubule [9], showing that the principal increase in the osmolarity of the fluid exiting the epithelium is established across the low‐resistance basement membrane of the lateral intercellular space. Because the primary tubular fluid is formed by ultrafiltration of plasma across the glomerular filtration barrier, hyperosmotic reabsorption of the primary filtrate by the proximal tubules (approximately 120 L daily in humans) would progressively raise extracellular fluid osmolarity, drive water out of cells, and rapidly disturb the normal distribution of fluid between extracellular and intracellular compartments. A conceptually simple way to achieve isosmotic reabsorption would be to recirculate the surplus ions back into the lateral intercellular space (Ussing) via a Na^+^ gradient‐driven co‐transporter in the cell membrane facing the serosal side of the epithelium [84]. To fulfill this purpose, we introduced a 1Na^+^:2Cl^−^:1 K^+^ cotransporter in the serosal cell membrane, consistent with experimental work in frog skin and small intestine [21, 84]. Controlled activation of this pathway enables isosmotic transport (Figure 6). As expected, this is at the expense of raised $C_{\mathrm{Cl}}^{c}$, here from 16.3 mM to 33.0 mM with an associated increase in cell volume from 1022 to 1667 nL·cm^−2^ (compare Figures 5 and 6). The cell volume of proximal straight tubule is physiologically regulated by VRAC [85], which upon activation by cell swelling causes intracellular Cl^−^ together with K^+^ to leave the cell with an associated decrease in cell water volume [86]. Our mathematical model of proximal tubule reduced $C_{\mathrm{Cl}}^{c}$ to 15.2 mM by “VRAC‐activation” together with an associated return of cell volume to 1013 nL·cm^−2^ (Figure 7). Interestingly, rabbit straight tubule perfused under light mineral oil at 38°C generated a hyperosmotic absorbate [87]. This finding is consistent with the interpretation that covering the external surface by oil interferes with the ion‐recirculation mechanism required for the normal conversion of hyperosmotic transport to isosmotic reabsorption.

### 3.4 Varying the Stoichiometry of H_2_O and Glucose of SGLT1 Indicates Cross Talk Between Water Flows via AQP and SGLT1

**FIGURE 8:**
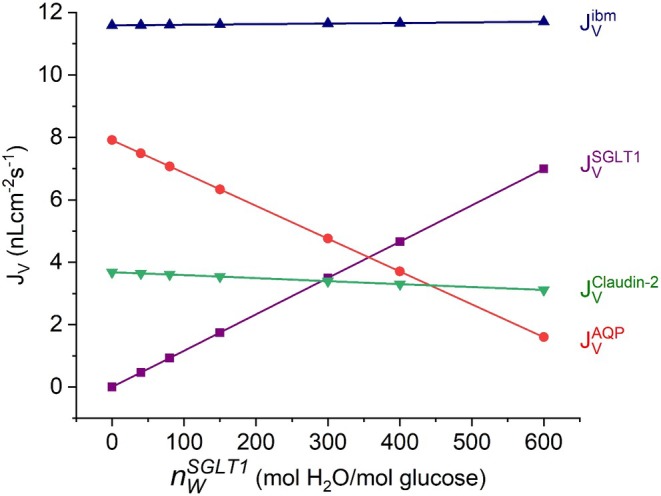
Computed relationships between the number of water molecules per glucose molecule transported by SGLT1 ($n_{W}^{\text{SGLT}1}$, Equation (3a)) and the water flow along the three transepithelial pathways, the two transcellular routes governed by AQP1 and SGLT1, respectively, and the paracellular route governed by Claudin‐2. Note the near‐constant fluid flow across the interspace basement membrane ($J_{V}^{\mathrm{ibm}}$) into the serosal bath, despite substantial variations in the water flow through the AQP1 and SGLT1 pathways. Se text for more details.

Water pathways of the S3‐segment of proximal tubule are governed by AQP1, the Na‐glucose cotransporter (SGLT1) both being expressed in the luminal membrane, and the junctional Claudin‐2. Below we pay special attention to the different mechanisms of water flow in these pathways. The number of water molecules associated the glucose uptake by SGLT1 ($n_{W}^{\text{SGLT}1}$ of Equation (3a)) is reported to vary, e.g., $n_{W}^{\text{SGLT}1}$ = 264 for human SGLT1 and 424 for rabbit SGLT1 [88]. Figure 8 shows computations of water flows across the model epithelium at varying $n_{W}^{\text{SGLT}1}$. The key result is that AQP1 mediated water uptake ($J_{V}^{\mathrm{AQP}}$) varies inversely with SGLT1‐associated water uptake ($J_{V}^{\text{SGLT}1}$), so that the total transepithelial water reabsorption indicated by $J_{V}^{\mathrm{ibm}}$ remains essentially unchanged even when water coupled to glucose via SGLT1 is varied from 0 to 600 mol H_2_O per mol glucose. The small decrease in the paracellular water uptake ($J_{V}^{\text{Claudin}-2}$) with decreasing $n_{W}^{\text{SGLT}1}$ is accompanied by minor changes in *V*_*t*_ and *Lis* ^*osm*^ (not shown) consistent with the essentially unchanged transepithelial water flux.

### 3.5 At Transepithelial Osmotic Equilibrium Is Fluid Uptake Energized by the Lateral Na^+^/K^+^‐Pump

**FIGURE 9:**
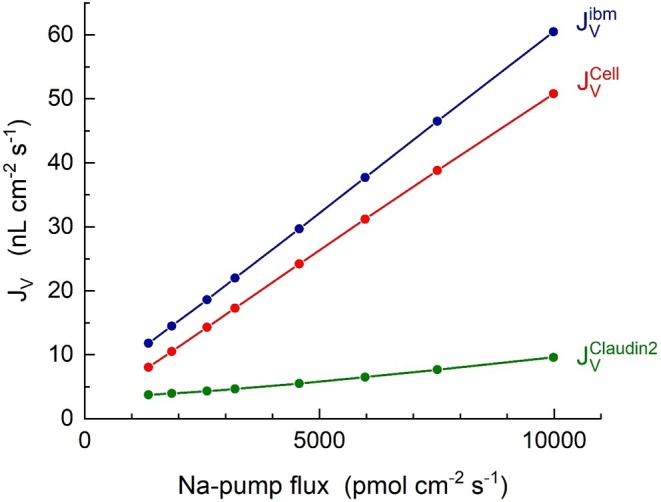
Computed dependence of fluid transport in the translateral ($J_{V}^{\mathrm{APQ}}+J_{V}^{\text{SGLT}1}=J_{V}^{\text{Cell}}$) and paracellular ($J_{V}^{\text{Cllaudin}2}$) pathways on the active Na^+^ flux generated by the lateral Na^+^/K^+^‐pump. Water flowing in the two translateral pathways governed by SGLT1 and AQP, are here added and indicated by $J_{V}^{\text{Cell}}$.

The results obtained above indicate that, at transepithelial osmotic equilibrium, the rate of fluid absorption is driven solely by the metabolic energy turnover associated with the activity of the lateral Na^+^/K^+^‐ATPase. This interpretation is supported by Figure 9, which shows that the model generates transepithelial water fluxes that are linearly dependent on the active sodium flux, $J_{\mathrm{Na}}^{\text{pump}}$. It is reassuring for our modeling approach that the above linear relationship between the rate of transepithelial water flux and the active Na^+^ flux reproduces previous in vitro findings obtained at transepithelial osmotic equilibrium in rat distal ileum [3], in Necturus proximal tubule preparations [5], and in amphibian skin epithelium [4].

### 3.6 The Metabolic Cost of Isosmotic Transport

According to our theory, isosmotic transport is achieved by recirculation of ions between the peritubular space and the lateral intercellular space (*lis*), driven by the lateral Na^+^/K^+^‐pump, which imposes a metabolic energy demand. This is conveniently expressed by the difference in the number of moles of ATP hydrolyzed before and after achieving isosmotic transport.

The computations shown in Table 2 confirm that isosmotic transport increases the energy metabolism expressed as mole ATP consumed. An additional result is that, as the number of moles of water associated glucose uptake via SGLT1 increases, the ion recirculation flux required for isosmotic transport decreases.

**TABLE 2:** Active sodium flux, fluid uptake, and the associated ATP hydrolysis before and after establishment of isosmotic transport by ion recirculation. The energetic cost of isosmotic transport, given as ΔATP in the last column, decreases as the number of water molecules associated with glucose flux via SGLT1 increases, $n_{W}^{\text{SGLT}1}=J_{W}^{\text{SGLT}1}/J_{\text{Gluc}}^{\text{SGLT}1}$ (Equation 3b).

$J_{W}^{\text{SGLT}1}/J_{\text{Gluc}}^{\text{SGLT}1}$	$J_{\mathrm{Na}}^{\text{active}}$	$J_{V}^{\mathrm{ibm}}$	ATP‐hydrolysed	*Isosmotic* $J_{\mathrm{Na}}^{\text{active}}$	Isosmotic $J_{V}^{\mathrm{ibm}}$	*Isosmotic* ATP hydrolysed	∆ATP
	pmolcm^−2^ s^−1^	nLcm^−2^ s^−1^	pmolcm^−2^ s^−1^	pmolcm^−2^ s^−1^	nLcm^−2^ s^−1^	pmolcm^−2^ s^−1^	pmolcm^−2^ s^−1^
0	1432	11.71	477.3	1622	12.85	540.6	63.3
200	1432	11.65	477.3	1613	12.74	537.6	60.3
400	1432	11.60	477.3	1603	12.64	534.3	57.0
600	1432	11.54	477.3	1594	12.53	531.3	54.0

## 4 Discussion

### 4.1 Electromotive Force of the Sodium Pump and Origin of Protons Driving Apical NHE3 at Reduced ΔG^ATP^

The electromotive force, *E* ^*pump*^ of the Na^+^/K^+^‐ATPase provides an electrochemical representation of the free energy of ATP hydrolysis at the pump site, thereby linking epithelial transport directly to cellular bioenergetics [11, 71]. In other words, the Na^+^/K^+^‐ATPase cannot generate ionic gradients exceeding those permitted by *E* ^*pump*^. In the present study, we apply the concept to interpret the profound changes in ion transport observed in isolated proximal tubules. As shown in Table 1, excised proximal tubule cells exhibit significant reduction in the cytosolic free energy of ATP hydrolysis (*ΔG* ^*ATP*^), accompanied by atypical intracellular cation concentrations, i.e., elevated Na^+^‐ and reduced K^+^‐concentration. Thus, the decrease in *ΔG* ^*ATP*^ directly translates into a reduced EMF of the Na^+^/K^+^‐ATPase, which imply weakening of the pumps ability to maintain steep transmembrane Na^+^ and K^+^ gradients. Under such conditions, conductive apical Na^+^ entry becomes energetically unfavorable, whereas Na^+^ uptake via electroneutral exchange mechanisms remains viable. The persistence of Na^+^ uptake via the apical Na^+^/H^+^ exchange mechanism (NHE3) necessarily implies a substantial and continuous proton supply. Under the restricted oxygen availability of isolated kidney tubules, mitochondrial oxidative phosphorylation cannot sustain normal phosphorylation potential, and the free energy of ATP hydrolysis available for the Na^+^/K^+^‐ATPase therefore diminishes. Thus, the reduced *E* ^*pump*^ reflects a shift in cellular energy metabolism toward non‐mitochondrial pathways. We therefore propose that the protons exchanged for Na^+^ via NHE3 originate predominantly from cytosolic metabolic processes associated with reduced *ΔG* ^*ATP*^, including enhanced glycolytic flux and ATP hydrolysis without efficient mitochondrial resynthesis. Within this thermodynamic context, NHE3 fulfills a dual role: it enables continued Na^+^ reabsorption despite a diminished *E* ^*pump*^ and simultaneously facilitates the extrusion of metabolically generated protons. Thus, the observed increase in proton secretion is not a primary regulatory response but a logical consequence of altered bioenergetic constraints. Viewed through the lens of Ussing's *E* ^*pump*^ concept, the repeatedly observed atypical intracellular cation concentrations in isolated proximal tubules are no longer paradoxical. Rather, they represent a coherent and predictable outcome of reduced pump electromotive force under in vitro conditions. This interpretation unifies altered energy metabolism, ion transport, and epithelial function within a single thermodynamic framework and highlights the continued relevance of the largely overlooked *E* ^*pump*^ concept for understanding renal epithelial physiology.

### 4.2 Transepithelial Water Reabsorption

The mechanism of isosmotic absorption by proximal tubule is a central theme in our most recent papers. To develop an understanding of fundamental principles, our studies focused on a minimalistic model, recognizing that a comprehensive description of kidney function would necessarily involve additional solutes, as demonstrated in recent studies from other laboratories [89, 90, 91, 92]. None of these studies assessed whether their modeled tubules generate isosmotic fluid absorption, which is central to renal homeostatic regulation. The analysis presented in the first part of this paper on fluid absorption as a simple consequence of active Na^+^ transport (Figures 4A–F and Figure 5), indicated that isosmotic transport cannot be obtained just by relying on the dissipative fluxes resulting from the activity of the lateral Na^+^/K^+^‐pump. Having developed an operational set of mathematical equations, we could pinpoint the forces responsible for driving water from the outside solution through the cell and the lateral intercellular space, and further, via the interspace basement membrane to the peritubular solution, revealing that the emerging hyperosmotic fluid in particular is the result of relatively large diffusion fluxes exiting the lateral intercellular basement membrane. This conclusion shows that isosmotic transport demands additional metabolic energy to bring the osmolarity of the transported fluid into equilibrium with the surrounding solutions. Our previous experimental studies of isosmotic transport in small intestinal and amphibian skin epithelia led us to hypothesize that the serosal 1Na^+^:2Cl^−^:1 K^+^ cotransporter, when properly adjusted, can fine‐tune the emerging fluid so that it becomes isosmotic [21, 84]. In the present computational study of the proximal tubule, it was indicated that regulated ion recirculation has capacity to fulfill the similar task in this low‐resistance epithelium (Figure 7).

A second focus was the interaction between the Na^+^/K^+^ pump in the lateral membrane and the two routes of water entry across the apical membrane, namely osmotic water flow through AQP1 and solute‐coupled water transport via SGLT1. Our integrated approach of mathematical modeling with computational analyses was applied to its full extent here. Firstly, a selective increase in water uptake through the SGLT1 pathway at constant active Na^+^ flux was accompanied by a corresponding decrease in water uptake through the AQP1 pathway; that is, the total translateral water flow remained the same (Figure 8). Secondly, the rate of water uptake was linearly dependent on the rate of active Na^+^ flux (Figure 9), reproducing experimental findings on net uptake of water in both low‐ and high‐resistance epithelia [3, 4, 5]. Thirdly, energized by the Na^+^/K^+^‐pump, isosmotic fluid reabsorption demands increased energy metabolism, expressed for example as moles of ATP hydrolysed. Importantly, the number of water molecules accompanying glucose uptake via SGLT1 reduces the hypertonicity generated by Na^+^ transport thereby decreases the amount of ion recirculation required; that is, SGLT1 indirectly lowers ATP consumption by the Na^+^/K^+^‐ATPase.

In conclusion, isosmotic reabsorption in the proximal tubule is an energy‐requiring process in which ATP‐dependent ion recirculation offsets the hypertonicity generated by active Na^+^ transport. Water transport associated with SGLT1‐mediated glucose uptake may slightly modify the energetic cost, but the dominant increased metabolic demand arises from Na^+^ transport itself.

## Author Contributions

**Erik Hviid Larsen:** conceptualization, funding acquisition, writing – original draft, methodology, validation, visualization, writing – review and editing, software, formal analysis, project administration, data curation, supervision, resources, investigation. **Jens Nørkær Sørensen:** conceptualization, investigation, funding acquisition, writing – original draft, methodology, validation, visualization, writing – review and editing, software, formal analysis, project administration, data curation, supervision, resources.

## Funding

The authors have nothing to report.

## Ethics Statement

The authors have nothing to report.

## Conflicts of Interest

The authors declare no conflicts of interest.

## Data Availability

All data supporting the findings of this study are given in the published text. The Fortran code is available upon request to the corresponding author.

## Appendix 1 Definitions and Symbols of Variables and Constants

**Table:**

Concentration of *j* (*Na* ^+^, *K* ^+^, *Cl* ^−^, *A* ^−^, *or glucose*) in compartment *comp* (*o*, *cell*, *lis or s*)	$C_{j}^{\text{comp}}$
Osmolarity of compartment indicated by superscript (*o, cell, lis or s*)	$\pi^{\text{comp}}$
Hydrostatic pressure of compartment indicated (*o, cell, lis or s*)	*p* ^*comp*^
Electrical potential of compartment indicated (*o, cell, lis or s*) with $\psi^{s}\equiv 0$	$\psi^{\text{comp}}$
Transepithelial potential difference, $\psi^{o}-\psi^{s}$	*V* ^*trans*^
Electrical potential difference between *lumen* and *cell*, $\psi^{o}-\psi^{c}$	*V* ^*am*^
Electrical potential difference between *cell* and *lis*, $\psi^{c}-\psi^{\mathrm{lis}}$	*V* ^*lm*^
Electrical potential difference between *cell* and *peritubular space*, $\psi^{c}-\psi^{s}$	*V* ^*c*^
Passive permeability of *j* in membrane *m* (*am, lm, pm, tj, ibm*)	$P_{j}^{m}$
Reflection coefficient of *j* (Na^+^, K^+^, Cl^−^, glucose) in *m* (*tj, ibm*)	$\sigma_{j}^{m}$
Flux of *j* (= Na^+^, K^+^, Cl^−^, glucose) across *m* (*am, lm, pm, tj, ibm*)	$J_{j}^{m}$
Electrical current carried by *j* (Na^+^, K^+^, Cl^−^) across *m* (*am, lm, pm, tj, ibm*)	$I_{j}^{m}$
Integral ion (*j*) conductance of membrane *m* (*am, lm, pm, tj, ibm*)	$G_{j}^{m}$
Water volume flux across *m* (*am, lm, pm, tj, ibm*)	$J_{V}^{m}$
Hydraulic conductance of membrane *m* (*am, lm, pm, tj, ibm*)	$L^{m}$
Osmotic permeability of *m* (*am, lm, pm, tj, ibm*)	$P_{f}^{m}$
Relative compliance constant of membrane *m* (*am, lm, pm*)	$\mu^{m}$
Absolute compliance constant of *lm*	${\bar{\mu }}^{\mathrm{lm}}$
Empirical constant of 1Na:1 K:2Cl cotransporter of membrane *m*	$K^{\mathrm{NaK}2\mathrm{Cl},m}$
Apparent dissociation constants of K^+^ binding of Na^+^/K^+^‐pump at *lm*	$K_{K}^{\text{pump},\mathrm{lm}}$
Empirical apparent dissociation constant of Na^+^:glucose transporter at *am*	$K_{\text{Gluc}}^{\text{Gluc}\cdot \mathrm{Na}}$
Empirical apparent dissociation constant for glucose of Na^+^:glucose transporter at *am*	$K_{\mathrm{Na}}^{\text{Gluc}\cdot \mathrm{Na}}$
Maximum turnover of glucose exchange mechanism at *lm*	$J_{\text{Gluc}}^{\max,\mathrm{lm}}$
App dissociation const. of symmetrical carrier at *lm*	$K_{\text{Gluc}}^{\mathrm{lm}}$
Turnover constant of 2 Na^+^:1 glucose transporter at *am*	$P^{\text{Gluc}\cdot \mathrm{Na}}$
Temperature in K	*T*
Faraday	*F*
Universal gas constant	*R*

## Appendix 2 Independent Variables of the Model's Standard Version

**Table:**

Name	Symbol	Value	MKSA‐unit
Hydraulic conductance of *am*	*L* ^*am*^	0.213d‐10	m^3^·s^−1^·*N* ^−1^
Hydraulic conductance of *pm*	*L* ^*pm*^	0.350d‐15	m^3^·s^−1^·*N* ^−1^
Hydraulic conductance of *lm*	*L* ^*lm*^	0.213d‐10	m^3^·s^−1^·*N* ^−1^
Hydraulic conductance of *tj*	*L* ^*tj*^	0.900d‐11	m^3^·s^−1^·*N* ^−1^
Hydraulic conductance of *ibm*	*L* ^*ibm*^	0.231d‐06	m^3^·s^−1^·*N* ^−1^
Na^+^ concentration of outside compartment	$C_{\mathrm{Na}}^{o}$	146	mol·m^−3^
K^+^ concentration of outside compartment	$C_{K}^{o}$	4	mol·m^−3^
Cl^−^ concentration of outside compartment	$C_{\mathrm{Cl}}^{o}$	150	mol·m^−3^
Glucose concentration of outside compartment	$C_{\text{Gluc}}^{o}$	6	mol·m^−3^
Na^+^ concentration of serosal compartment	$C_{\mathrm{Na}}^{s}$	146	mol·m^−3^
K^+^ concentration of serosal compartment	$C_{K}^{s}$	4	mol·m^−3^
Cl^−^ concentration of serosal compartment	$C_{\mathrm{Cl}}^{s}$	150	mol·m^−3^
Glucose concentration of serosal	$C_{\text{Gluc}}^{s}$	6	mol·m^−3^
Na^+^ GHK permeability of *am*	$P_{\mathrm{Na}}^{\mathrm{am}}$	0.200d‐7	m·s^−1^
K^+^ GHK permeability of *am*	$P_{K}^{\mathrm{am}}$	0.100d‐13	m·s^−1^
Cl^−^ GHK permeability of *am*	$P_{\mathrm{Cl}}^{\mathrm{am}}$	0.100d‐13	m·s^−1^
Na^+^ GHK permeability of *pm*	$P_{\mathrm{Na}}^{\mathrm{pm}}$	0.100d‐12	m·s^−1^
K^+^ GHK permeability of *pm*	$P_{K}^{\mathrm{pm}}$	0.100d‐12	m·s^−1^
Cl^−^ GHK permeability of *pm*	$P_{K}^{\mathrm{pm}}$	0.100d‐12	m·s^−1^
Na^+^ GHK permeability of *lm*	$P_{\mathrm{Na}}^{\mathrm{lm}}$	0.300d‐14	m·s^−1^
K^+^ GHK permeability of *lm*	$P_{K}^{\mathrm{lm}}$	0.500d‐6	m·s^−1^
Cl^−^ GHK permeability of *lm*	$P_{\mathrm{Cl}}^{\mathrm{lm}}$	0.700d‐7	m·s^−1^
Na^+^ GHK permeability of *tj*	$P_{\mathrm{Na}}^{\mathrm{tj}}$	0.600d‐7	m·s^−1^
K^+^ GHK permeability of *tj*	$P_{K}^{\mathrm{tj}}$	0.612d‐7	m·s^−1^
Cl^−^ GHK permeability of *tj*	$P_{\mathrm{Cl}}^{\mathrm{tj}}$	0.270d‐5	m·s^−1^
Glucose permeability of *tj*	$P_{\text{Gluc}}^{\mathrm{tj}}$	0.500d‐9	m·s^−1^
Na^+^ GHK permeability of *ibm*	$P_{\mathrm{Na}}^{\mathrm{ibm}}$	0.120d‐5	m·s^−1^
K^+^ GHK permeability of *ibm*	$P_{K}^{\mathrm{ibm}}$	0.176d‐5	m·s^−1^
Cl^−^ GHK permeability of *ibm*	$P_{\mathrm{Cl}}^{\mathrm{ibm}}$	0.350d‐4	m·s^−1^
Glucose permeability if *ibm*	$P_{\text{Gluc}}^{\mathrm{ibm}}$	0.350d‐5	m·s^−1^
1Na:2Cl:1 K cotransporter constant of *am*	*K* ^*Na2ClK,am*^	0.28d‐13	m^10^·s^−1^·mol^−3^
1Na:2Cl:1 K cotransporter constant of *pm*	*K* ^*Na2ClK,pm*^	Dependent	m^10^·s^−1^·mol^−3^
Turnover constant of lateral 3Na^+^/2 K^+^ pump	$P_{\mathrm{Na},K}^{\mathrm{lm},\text{pump}}$	0.350d‐3	mol·m^−2^·s^−1^
Electromotive force of lateral 3Na^+^/2 K^+^ pump	*E* ^*pump*^	0.200	volt
Reversal potential of lateral 3Na^+^/2 K^+^ pump	$V_{\mathrm{rev}}^{\mathrm{lm},\text{pump}}$	−0.200	volt
Apparent dissociation constants of Na^+^ binding	$K_{\mathrm{Na}}^{\text{pump},\mathrm{lm}}$	3.40d0	mol·m^−3^
Apparent dissociation constants of K^+^ binding	$K_{K}^{\text{pump},\mathrm{lm}}$	0.75d0	mol·m^−3^
Turnover constant of SGLT1 at *am*	*P* ^*SGLT1,max*^	0.70d‐12	mol·m^−2^·s^−1^
Apparent dissociation constant for glucose at SGLT1	*K* ^*SGLT1*^	1.0	mol·m^−3^
Maximum turnover of glucose transporter (GLUT) *lm*	*J* ^*Gluc‐max,lm*^	0.10d‐3	mol·m^−2^·s^−1^
App dissociation const. of symmetry carrier at *lm*	$K_{\mathrm{Glu}}^{\mathrm{lm}}$	5.0	mol·m^−3^
Hydrostatic pressure of outer (luminal) compartment	*p* ^*o*^	101325e5	Pa
Hydrostatic pressure of peritubular compartment	*p* ^*ps*^	101325e5	Pa
Mean valence of nondiffusible intracellular anions	*z*_*A*_	−0.95	
Na^+^ reflection coefficient of *tj*	$\sigma_{\mathrm{Na}}^{\mathrm{tj}}$	0.70	
K^+^ reflection coefficient of *tj*	$\sigma_{K}^{\mathrm{tj}}$	0.70	
Cl^−^ reflection coefficient of *tj*	$\sigma_{\mathrm{Cl}}^{\mathrm{tj}}$	0.45	
Glucose reflection coefficient of *tj*	$\sigma_{\text{Gluc}}^{\mathrm{tj}}$	0.80	
Na^+^ reflection coefficient of *ibm*	$\sigma_{\mathrm{Na}}^{\mathrm{ibm}}$	0.03	
K^+^ reflection coefficient of *ibm*	$\sigma_{K}^{\mathrm{ibm}}$	0.03	
Cl^−^ reflection coefficient of *ibm*	$\sigma_{\mathrm{Cl}}^{\mathrm{ibm}}$	0.03	
Glucose reflection coefficient of *ibm*	$\sigma_{\text{Gluc}}^{\mathrm{ibm}}$	0.03	
Temperature	T	310	K
Faraday	*F*	96 485	C·mol^−1^
Absolute compliance constant of *lm*	*μ* ^*lm*^	0.250d‐2	Pa^−1^
Reference volume of *lis*	*Vol* ^lis,ref^	3.10d‐7	m^3^·m^−2^
Cell density	*D* ^*c*^	4.00d + 9	# cells·m^−2^
Non‐diffusible anions in cell	*M*_*A*_	3.50d‐13	mol·cell^−1^
